# Omeprazole and Lansoprazole Enantiomers Induce CYP3A4 in Human Hepatocytes and Cell Lines via Glucocorticoid Receptor and Pregnane X Receptor Axis

**DOI:** 10.1371/journal.pone.0105580

**Published:** 2014-08-20

**Authors:** Aneta Novotna, Zdenek Dvorak

**Affiliations:** Regional Centre of Advanced Technologies and Materials, Faculty of Science, Palacky University, Olomouc, Czech Republic; The University of Iowa, United States of America

## Abstract

Benzimidazole drugs lansoprazole and omeprazole are used for treatment of various gastrointestinal pathologies. Both compounds cause drug-drug interactions because they activate aryl hydrocarbon receptor and induce CYP1A genes. In the current paper, we examined the effects of lansoprazole and omeprazole enantiomers on the expression of key drug-metabolizing enzyme CYP3A4 in human hepatocytes and human cancer cell lines. Lansoprazole enantiomers, but not omeprazole, were equipotent inducers of CYP3A4 mRNA in HepG2 cells. All forms (S-, R-, rac-) of lansoprazole and omeprazole induced CYP3A4 mRNA and protein in human hepatocytes. The quantitative profiles of CYP3A4 induction by individual forms of lansoprazole and omeprazole exerted enantiospecific patterns. Lansoprazole dose-dependently activated pregnane X receptor PXR in gene reporter assays, and slightly modulated rifampicin-inducible PXR activity, with similar potency for each enantiomer. Omeprazole dose-dependently activated PXR and inhibited rifampicin-inducible PXR activity. The effects of S-omeprazole were much stronger as compared to those of R-omeprazole. All forms of lansoprazole, but not omeprazole, slightly activated glucocorticoid receptor and augmented dexamethasone-induced GR transcriptional activity. Omeprazole and lansoprazole influenced basal and ligand inducible expression of tyrosine aminotransferase, a GR-target gene, in HepG2 cells and human hepatocytes. Overall, we demonstrate here that omeprazole and lansoprazole enantiomers induce CYP3A4 in HepG2 cells and human hepatocytes. The induction comprises differential interactions of omeprazole and lansoprazole with transcriptional regulators PXR and GR, and some of the effects were enantiospecific. The data presented here might be of toxicological and clinical importance, since the effects occurred in therapeutically relevant concentrations.

## Introduction

Many clinically used drugs contain in their chemical structure chiral atom, therefore, they exist in 2^n^ conformations – enantiomers, where *n* stands for number of chiral centers in the molecule. Individual enantiomers may have qualitatively (e.g. different cellular targets) and quantitatively (e.g. different EC_50_, IC_50_, K_D_, K_M_ etc.) different pharmacokinetic and pharmacodynamics properties. Enantiomer with high (or desired) and low (or undesired) therapeutic activity is called *eutomer* and *dystomer*, respectively. The ratio between pharmacokinetic parameters of eutomer and dystomer is called *eudysmic ratio*. The facts that *eudysmic ratio* is largely different from “1” justifies the use of enantiopure drugs in clinical practice, which was also the case of benzimidazole proton pump inhibitors omeprazole (OME) and lansoprazole (LAN). Both compounds contain the asymmetric chiral sulfur atom in their chemical structure and therefore they exist in form R- and S-enantiomers. Enantiopure drug Esomeprazole (S-OME), having improved metabolic properties, such as higher bioavailability and lower inter-individual variation as compared to racemic drug was developed in 2001 [1]–[3]. FDA has approved Dexlansoprazole (R-LAN) in 2009 as an enatiopure drug for treatment of gastro esophageal reflux disease [4], [5].

Drug-drug interactions or drug adverse effects may occur when a drug is an inducer of drug-metabolizing enzymes. It is well known that omeprazole and lansoprazole are inducers of CYP1A1 and CYP1A2 enzymes, which are involved in xenobiotics metabolism and chemically induced carcinogenesis. The induction is mediated through aryl hydrocarbon receptor (AhR), but OME and LAN are not ligands for AhR [6]. We have recently demonstrated that the effects of OME and LAN on AhR-CYP1A signaling pathway are enantiospecific [7]. There are several reports that omeprazole is an inducer of human CYP3A4, an enzyme involved in metabolism of over 60% of known drugs. Cell-based reporter gene assay in HepG2 cells showed an induction of CYP3A4-mediated luciferase activity by omeprazole [8], [9]. Omeprazole induced CYP3A4 mRNA expression (4-fold by 100 µM–200 µM OME) in primary human hepatocytes [10]. Main transcriptional regulators of CYP3A4 are pregnane X receptor (PXR) and glucocorticoid receptor (GR), but other receptors such as vitamin D receptor, constitutive androstane receptor and others are involved in CYP3A4 regulation [11].

In the current paper we examined the effects of lansoprazole and omeprazole enantiomers on the expression of CYP3A4 in human hepatocytes and human cancer cell lines, and on transcriptional activity of PXR and GR in transgenic cell lines. We demonstrate that omeprazole and lansoprazole enantiomers induce CYP3A4 and that the induction comprises differential interactions of omeprazole and lansoprazole with transcriptional regulators PXR and GR, and some of the effects are enantiospecific. The data presented here might be of toxicological and clinical importance.

## Materials and Methods

### Compounds and reagents

Dimethylsulfoxide (DMSO), rifampicin (RIF), dexamethasone (DEX), mifepristone (RU486) and hygromycin B were purchased from Sigma-Aldrich (Prague, Czech Republic). S-omeprazole (S-OME), R-omeprazole (R-OME), rac-omeprazole (rac-OME), S-lansoprazole (S-LAN), R-lansoprazole (R-LAN) and rac-lansoprazole (rac-LAN) were purchased from Santa Cruz Biotechnology Inc. (Heidelberg, Germany). Luciferase lysis buffer was from Promega (Hercules, CA).

### Cell culture

Human Caucasian colon adenocarcinoma cells LS174T (ECACC No. 87060401) and human Caucasian hepatocellular carcinoma cells HepG2 (ECACC No. 85011430) were purchased from ECACC and were cultured in as recommended by manufacturer. Primary human hepatocytes used in this study were obtained from two sources: (i) from multiorgan donor HH52 (female; 60 years); the use of liver cells of donor HH52 was approved by “Ethical committee at the Faculty Hospital Olomouc”, and it was in accordance with Transplantation law #285/2002 Sb; “Ethical committee at the Faculty Hospital Olomouc” waived the authors from obtaining consent from the next of kin, regarding human hepatocytes obtained from liver donor HH52. (ii) long-term human hepatocytes in monolayer Batch HEP220770 (female; 35 years) were purchased from Biopredic International (Biopredic International, Rennes, France). Cells were cultured in serum-free medium. Cultures were maintained at 37°C and 5% CO_2_ in a humidified incubator.

### mRNA determination and quantitative reverse transcriptase polymerase chain reaction

Total RNA was isolated using TRI Reagent (Molecular Research Center, Cincinnati, OH, USA). cDNA was synthesized from 1000 ng of total RNA using M-MLV Reverse Transcriptase (Finnzymes, Espoo, Finland) at 42°C for 60 min in the presence of random hexamers (Takara, Shiga, Japan). qRT-PCR was carried out using LightCycler FastStart DNA MasterPLUS SYBR Green I (Roche Diagnostic Corporation, Prague, Czech Republic) on a Light Cycler 480 II apparatus (Roche Diagnostic Corporation). CYP3A4, TAT and GAPDH mRNAs were determined as described previously [12]. Measurements were performed in triplicates. Gene expression was normalized to GAPDH as a housekeeping gene.

### Protein detection and Western blotting

Total protein extracts were prepared as described elsewhere [7]. SDS–PAGE gels (10%) were run on a BioRad apparatus according to the general procedure followed by the protein transfer onto PVDF membrane. The membrane was saturated with 5% non-fat dried milk for 1 h at room temperature. Blots were probed with primary antibodies against CYP3A4 (mouse monoclonal; sc-53850, HL3) and actin (goat polyclonal; sc-1616, 1–19), both purchased from Santa Cruz Biotechnology (Santa Cruz, CA, USA). Chemiluminescent detection was performed using horseradish peroxidase-conjugated secondary antibodies (Santa Cruz Biotechnology) and Western blotting Luminol kit (Santa Cruz Biotechnology). The density of bands was measured by densitometry.

### Gene reporter assay and cytotoxicity assay

A stably transfected gene reporter cell line AZ-GR was used for assessment of GR transcriptional activity [13]. A transiently transfected LS174T human colon adenocarcinoma cells were used for assessment of PXR transcriptional activity. A chimera *p3A4-luc* reporter construct containing the basal promoter (−362/+53) with proximal PXR response element and the distal xenobiotic responsive enhancer module (−7836/−7208) of the *CYP3A4* gene 5′-flanking region inserted to pGL3-Basic reporter vector was used. The reporter plasmid was transiently transfected to LS174T cells by lipofection (FuGENE 6) with 300 ng/well of *p3A4-luc* reporter in 24-well plates. Cells were incubated for 24 h with tested compounds and/or vehicle (DMSO; 0.1% v/v), in the presence or absence of RIF (10 µM; LS174T cells) or DEX (100 nM; AZ-GR cells). After the treatments, cells were lysed and luciferase activity was measured. In parallel, cell viability was determined by conventional MTT test.

### Statistics

Experiments in cell cultures were performed at least in three different cell passages. In each passage, treatments of cells were performed in triplicates. For measurement of luminescence (luciferase activity) and absorbance (MTT), triplicates from each sample were run. One-way analysis of variance followed by Dunnett’s multiple comparison post hoc test or Student’s *t* test was used for statistical analysis of data.

## Results

### Effects of omeprazole and lansoprazole enantiomers on CYP3A4 mRNA and protein expression in human cancer cell lines and human hepatocytes

**Figure 1:**
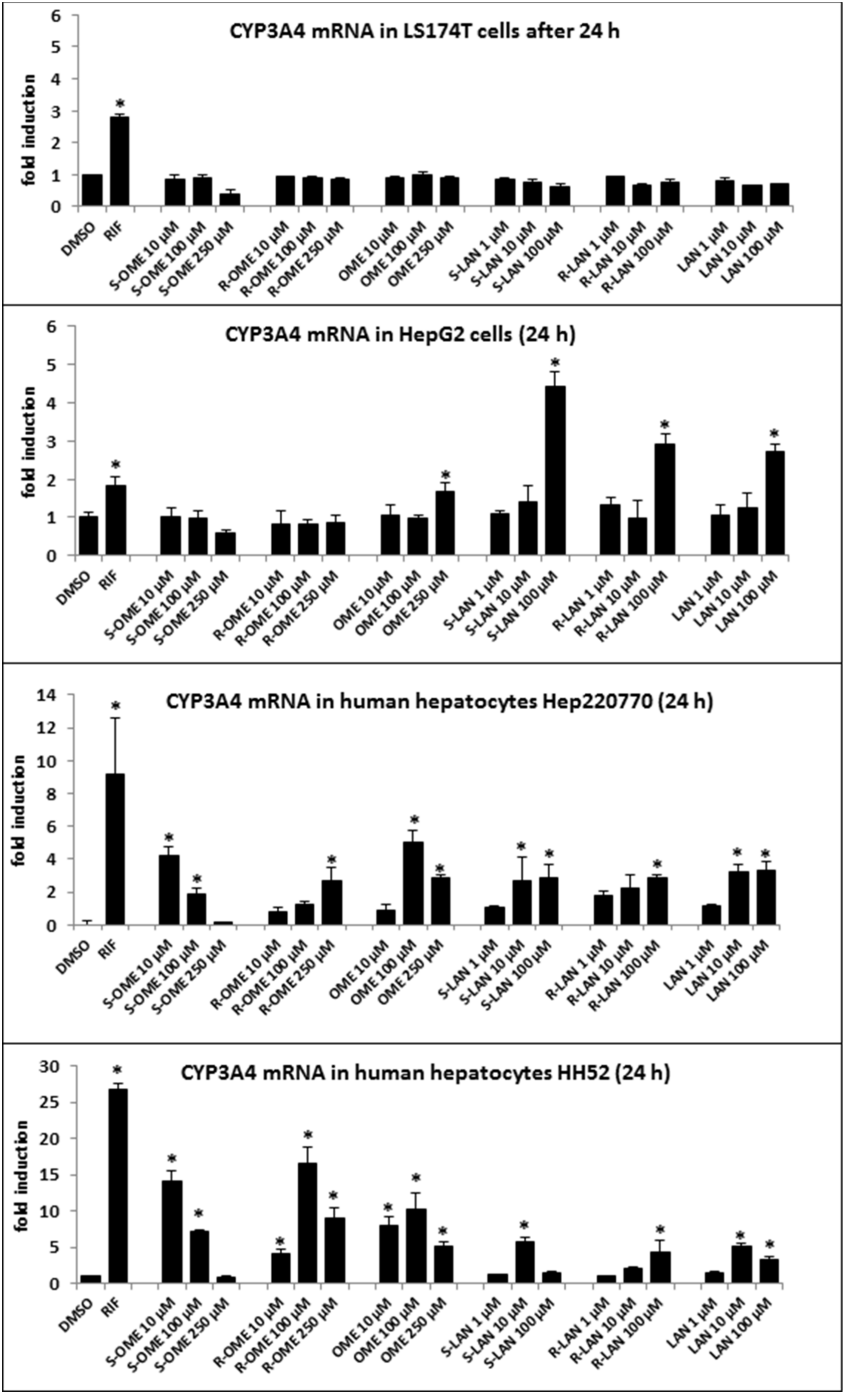
Effects of omeprazole and lansoprazole enantiomers on CYP3A4 mRNA expression in human cancer cell lines and human hepatocytes. (i) HepG2 and LS174T cells were seeded in 6-well plates and stabilized for 16 h. All experiments were performed in three consecutive cell passages. (ii) Primary human hepatocytes from two different donors (HH52 and Hep220770) were used. Cells were incubated for 24 h with RIF (10 µM), vehicle (DMSO; 0.1% v/v), omeprazole (S-, R-, rac-; 10 µM, 100 µM, 250 µM) and lansoprazole (S-, R-, rac-; 1 µM, 10 µM, 100 µM). Representative RT-PCR analyses of CYP3A4 mRNA are shown. The data are the mean ± SD from triplicate measurements and are expressed as a fold induction over vehicle-treated cells. The data were normalized to GAPDH mRNA levels. An asterisk (*) indicates that the value is significantly different from the activity of vehicle-treated cells.

**Figure 2:**
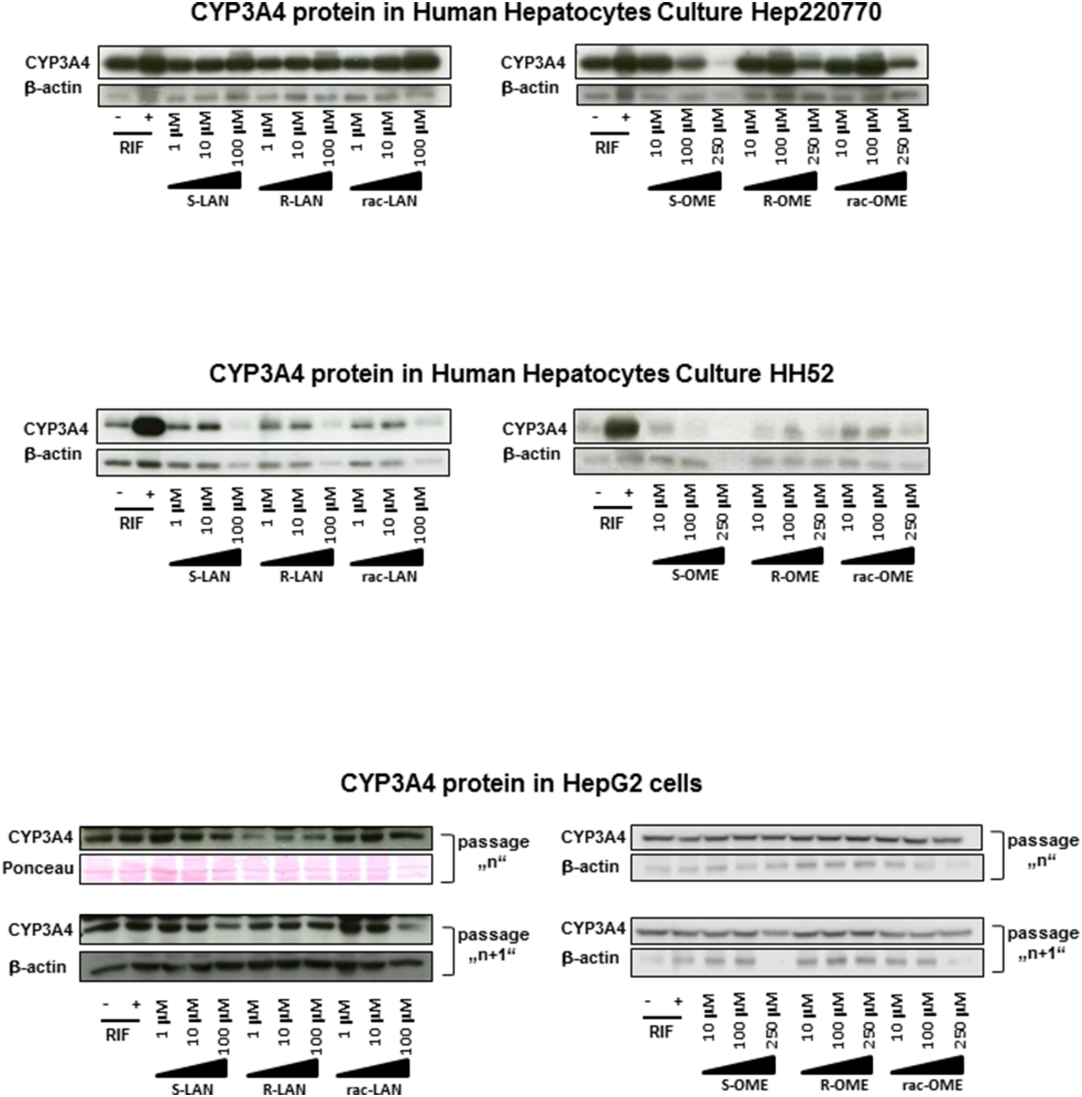
Effects of omeprazole and lansoprazole enantiomers on CYP3A4 protein expression in HepG2 cells and human hepatocytes. Western blots of CYP3A4 and β-actin from two different human hepatocytes cultures (HH52 and Hep220770) and from two consecutive passages of HepG2 cells are shown. Cells were incubated for 48 h with RIF (10 µM), vehicle (DMSO; 0.1% v/v), omeprazole (S-, R-, rac-; 10 µM, 100 µM, 250 µM) and lansoprazole (S-, R-, rac-; 1 µM, 10 µM, 100 µM). Density of bands was quantified by densitometry. An asterisk (*) indicates that the value is significantly different from the activity of DMSO.

In the first series of experiments, we tested the ability of omeprazole and lansoprazole enantiomers to induce the expression of CYP3A4. Human hepatoma HepG2 cells, intestinal cancer cells LS174T and primary human hepatocytes were treated with rifampicin (RIF; 10 µM), vehicle (DMSO; 0.1% V/V), S-OME, R-OME, rac-OME, S-LAN, R-LAN and rac-LAN at concentrations ranging from 1 µM to 250 µM for 24 h (mRNA expression) and 48 h (protein expression). Rifampicin, a model activator of PXR and an inducer of CYP3A4 induced CYP3A4 mRNA by factors 2-fold, 3-fold, 9-fold and 27-fold in LS174T cells, HepG2 cells, hepatocytes culture Hep2220770 and hepatocytes culture HH52 as compared to vehicle-treated cells, respectively. Significant induction of CYP3A4 in HepG2 cells was observed for rac-OME (250 µM; 2-fold) and all forms of LAN in 100 µM concentration (3–5 fold) (Figure 1). Consistently, LAN induced CYP3A4 protein in HepG2 cells, with strongest effects observed for S-LAN, while there was no induction by any form of OME (Figure 2). Interestingly, there was no induction of CYP3A4 mRNA in LS174T cells by any form of OME or LAN in any concentration. We did not measure the expression of CYP3A4 protein in LS174T cells, since CYP3A4 protein is expressed constitutively and it is not inducible by xenobiotics. All forms (S-, R-, rac-) of OME and LAN induced CYP3A4 mRNA and protein in two human hepatocytes cultures. The quantitative profiles of CYP3A4 induction by individual forms of LAN and OME exerted enantiospecific patterns (Figure 1, Figure 2). Overall, some of the effects of omeprazole and lansoprazole on CYP3A4 mRNA and protein expression in HepG2 cells and human hepatocytes were enantiospecific.

### Effects of omeprazole and lansoprazole enantiomers on transcriptional activity of pregnane X receptor PXR in human LS174T gene reporter cell lines

**Figure 3:**
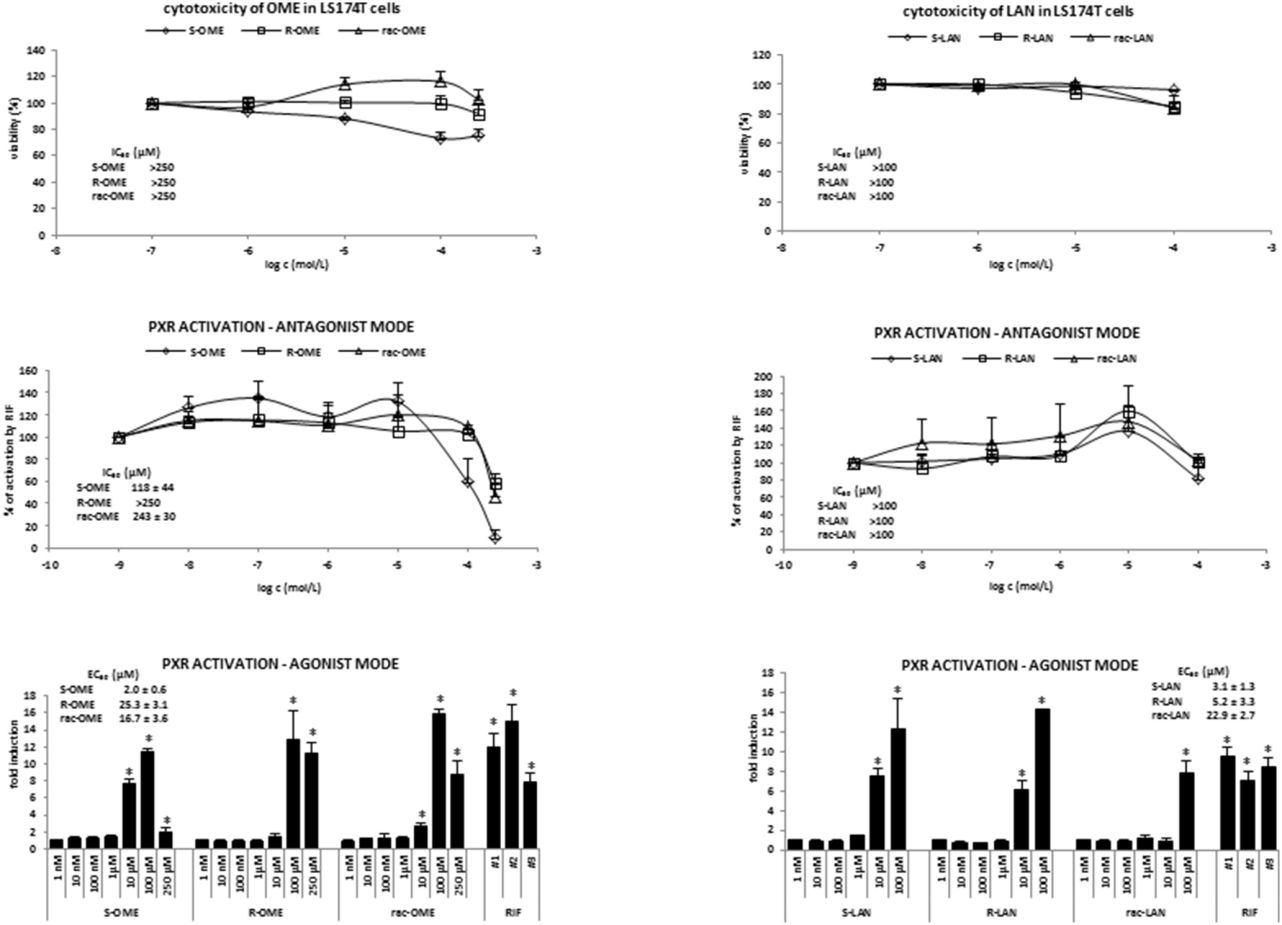
Effect of omeprazole and lansoprazole enantiomers on transcriptional activity of pregnane X receptor PXR in transiently transfected LS174T cells. LS174T cells, transiently transfected with *p3A4-luc* reporter, were seeded in 24-well plates, stabilized for 16 h and then incubated for 24 h with S-OME, R-OME, rac-OME, S-LAN, R-LAN and rac-LAN at concentrations ranging from 1 nM to 250 µM. The vehicle was DMSO (0.1% v/v). Model activator of PXR was rifampicin (RIF; 10 µM). Treatments were performed in triplicates. ***Upper panels:*** MTT test was performed and absorbance was measured at 540 nm. The data are the mean from experiments from three consecutive passages of cells and are expressed as a percentage of viability of control cells. The values of IC_50_ were calculated and are indicated in a figure. ***Middle panels:*** *Antagonist mode -* Transfected LS174T cells were incubated with OME and LAN in the presence of RIF (10 µM). The data are the mean from experiments from four consecutive passages of cells and are expressed as a percentage of maximal induction attained by RIF. The values of IC_50_ were calculated and the average values are indicated in figures. ***Lower panels:*** *Agonist mode* - Transfected LS174T cells were incubated with OME and LAN in the absence of RIF (10 µM). The data are the mean from experiments from four consecutive passages of cells and are expressed as a fold induction of luciferase activity over control cells. The values of EC_50_ and were calculated and the average values are indicated in figures.

In next series of experiments, the effects of OME and LAN on transcriptional activity of PXR, a key regulator of CYP3A4, were assessed in human colon adenocarcinoma cells LS174T transiently transfected with *p3A4-luc* reporter construct (for details see Materials and Methods section). Prior to the gene reporter assays, cytotoxicity of tested compounds in LS174T cells after 24 h of incubation was assessed by conventional MTT test (cytotoxicity of OME and LAN in HepG2 cells and human hepatocytes was tested elsewhere [7]). We did not observe significant decline in viability of LS174T cells by LAN (up to 100 µM) and OME (up to 250 µM) (Figure 3; upper panels). Gene reporter assays were performed in two different experimental layouts. In *agonist mode*, cells were treated with increasing concentrations of LAN and OME, and the half-maximal effective concentrations (EC_50_) were calculated. In *antagonist mode*, cells were incubated with increasing concentrations of tested compounds in combination with model PXR agonist rifampicin (RIF; 10 µM), and half-maximal inhibitory concentrations (IC_50_) were calculated, where appropriate. An induction of PXR-dependent luciferase activity by rifampicin varied from 7-fold to 15-fold, as compared to vehicle-treated cells. Both enantiomers of OME and LAN strongly activated PXR, with fold inductions comparable to those by rifampicin (Figure 3; bottom panels). The effects of S-LAN (EC_50_ = 3.1±1.3 µM) and R-LAN (EC_50_ = 5.2±3.3 µM) were not enantiospecific, and the induction was significant in concentrations 10 µM and 100 µM. On the other hand, the activation patterns of PXR by S-OME and R-OME differed between enantiomers. R-OME strongly activated PXR in concentrations 100 µM and 250 µM (EC_50_ = 25.3±3.1 µM). S-OME activated PXR in concentrations 10 µM and 100 µM with drop of activation at 250 µM (EC_50_ = 2.0±0.6 µM). Racemic forms of OME and LAN displayed somehow mixed effects of S- and R- enantiomers. In antagonist mode, all forms of LAN in concentration 10 µM systematically augmented activation of PXR by rifampicin up to 120%–140% of control value. However, in concentration of LAN 100 µM the PXR activation dropped back to 90%–100% of initial value attained by rifampicin in the absence of LAN. All forms of OME dose-dependently inhibited rifampicin-induced activity of PXR. The effects of S-OME were much stronger as compared to those of R-OME (Figure 3; middle panels). Collectively, both OME and LAN influenced basal and ligand-activated PXR transcriptional activity. The effects of OME but not LAN were enantiospecific.

### Effects of omeprazole and lansoprazole enantiomers on transcriptional activity of glucocorticoid receptor GR in AZ-GR gene reporter cell line

**Figure 4:**
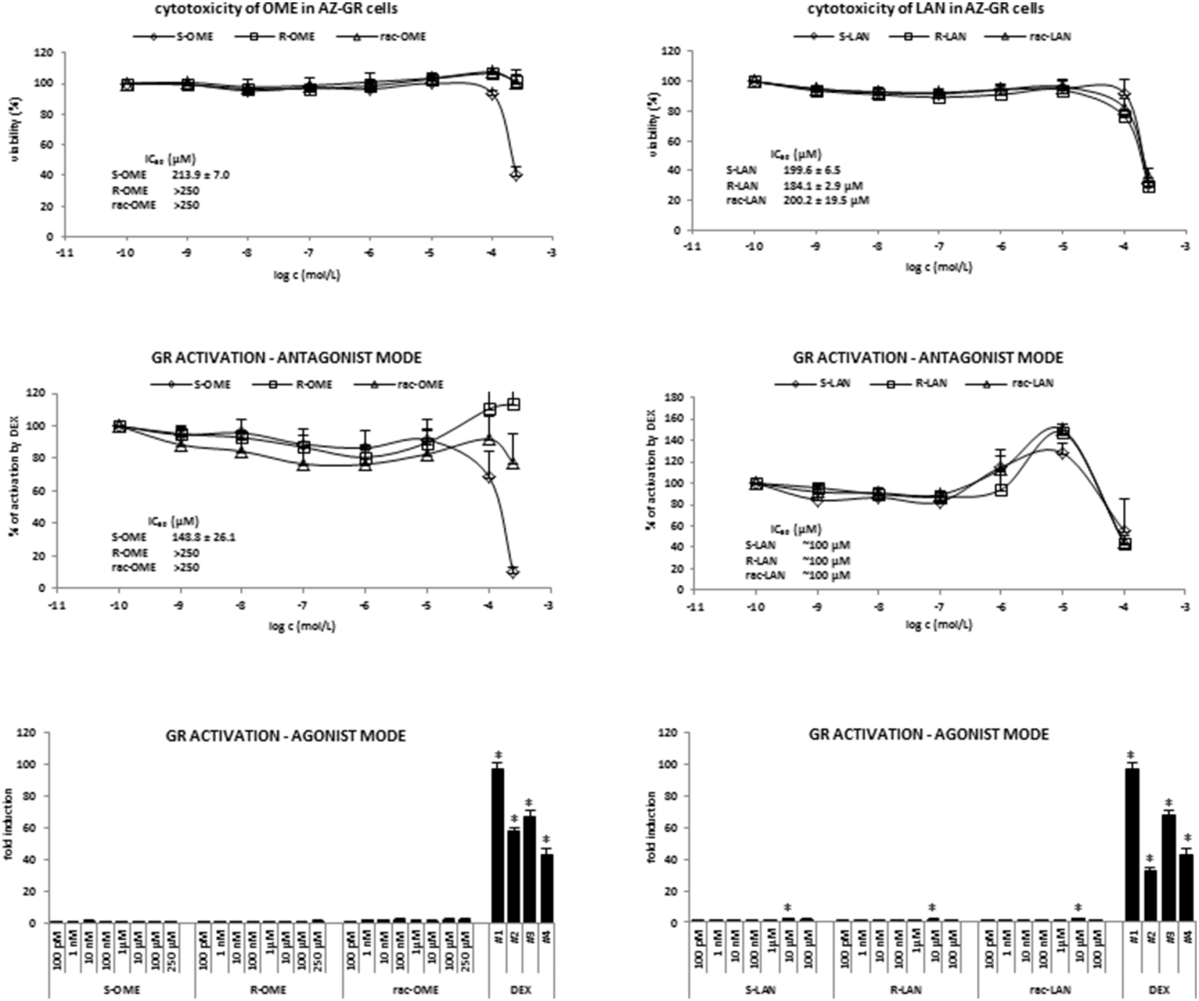
Effect of omeprazole and lansoprazole enantiomers on transcriptional activity of glucocorticoid receptor GR in AZ-GR transgenic cells. AZ-GR cells were seeded in 96-well plates, stabilized for 16 h and then incubated for 24 h with S-OME, R-OME, rac-OME, S-LAN, R-LAN and rac-LAN at concentrations ranging from 0.1 nM to 250 µM. The vehicle was DMSO (0.1% v/v). Model activator of GR was dexamethasone (DEX; 0.1 µM). Treatments were performed in triplicates. ***Upper panels:*** MTT test was performed and absorbance was measured at 540 nm. The data are the mean from experiments from three consecutive passages of cells and are expressed as a percentage of viability of control cells. The values of IC_50_ were calculated and are indicated in a figure. ***Middle panels:*** *Antagonist mode –* AZ-GR cells were incubated with OME and LAN in the presence of DEX (0.1 µM). The data are the mean from experiments from four consecutive passages of cells and are expressed as a percentage of maximal induction attained by RIF. The values of IC_50_ were calculated and the average values are indicated in figures. ***Lower panels:*** *Agonist mode* - Transfected LS174T cells were incubated with OME and LAN in the absence of DEX. The data are the mean from experiments from four consecutive passages of cells and are expressed as a fold induction of luciferase activity over control cells. The values of EC_50_ and were calculated and the average values are indicated in figures.

The effects of OME and LAN enantiomers on transcriptional activity of GR, a pivotal regulator of drug-metabolizing enzymes, were assessed in transgenic reporter cell line AZ-GR. As revealed by MTT test in AZ-GR cells incubated for 24 h with tested compounds, both OME and LAN displayed dose-dependent cytotoxicity against this cell line. The cytotoxicity of LAN enantiomers did not significantly differ from each other, with IC_50_ values ranging around 200 µM. Interestingly, S-OME (IC_50_ = 214 µM) was significantly more cytotoxic as compared to R-OME enantiomer (Figure 4, upper panels). An induction of GR-dependent luciferase activity by synthetic glucocorticoid dexamethasone varied from 27-fold to 97-fold, as compared to vehicle-treated cells. Lansoprazole but not omeprazole had weak effects on basal activity of GR, with significant increase of luciferase activity in cells incubated with all forms of LAN (10 µM). However, this induction was less than 5% of induction attained by DEX (Figure 4; lower panels). In antagonist mode, OME had not inhibitory effects on DEX-induced GR transcriptional activity; strong decrease of luciferase activity in 250 µM concentrations of OME is consistent with cytotoxicity data. All forms of LAN augmented DEX-induced GR transcriptional activity in 10 µM concentration (up to 130%–140% of initial value), while there was a drop in luciferase activity in 100 µM concentrations of LAN, which is due to the cytotoxic effects (Figure 4; middle panels). Overall, LAN and OME were cytotoxic to AZ-GR cells, and effects of OME but not LAN were enantiospecific. Lansoprazole but not omeprazole exerted partial agonist activity towards GR, i.e. it slightly increased basal and ligand inducible GR transcriptional activity. These effects were not enantiospecific.

### Effects of omeprazole and lansoprazole enantiomers on TAT mRNA expression in HepG2 cells and primary human hepatocytes

**Figure 5:**
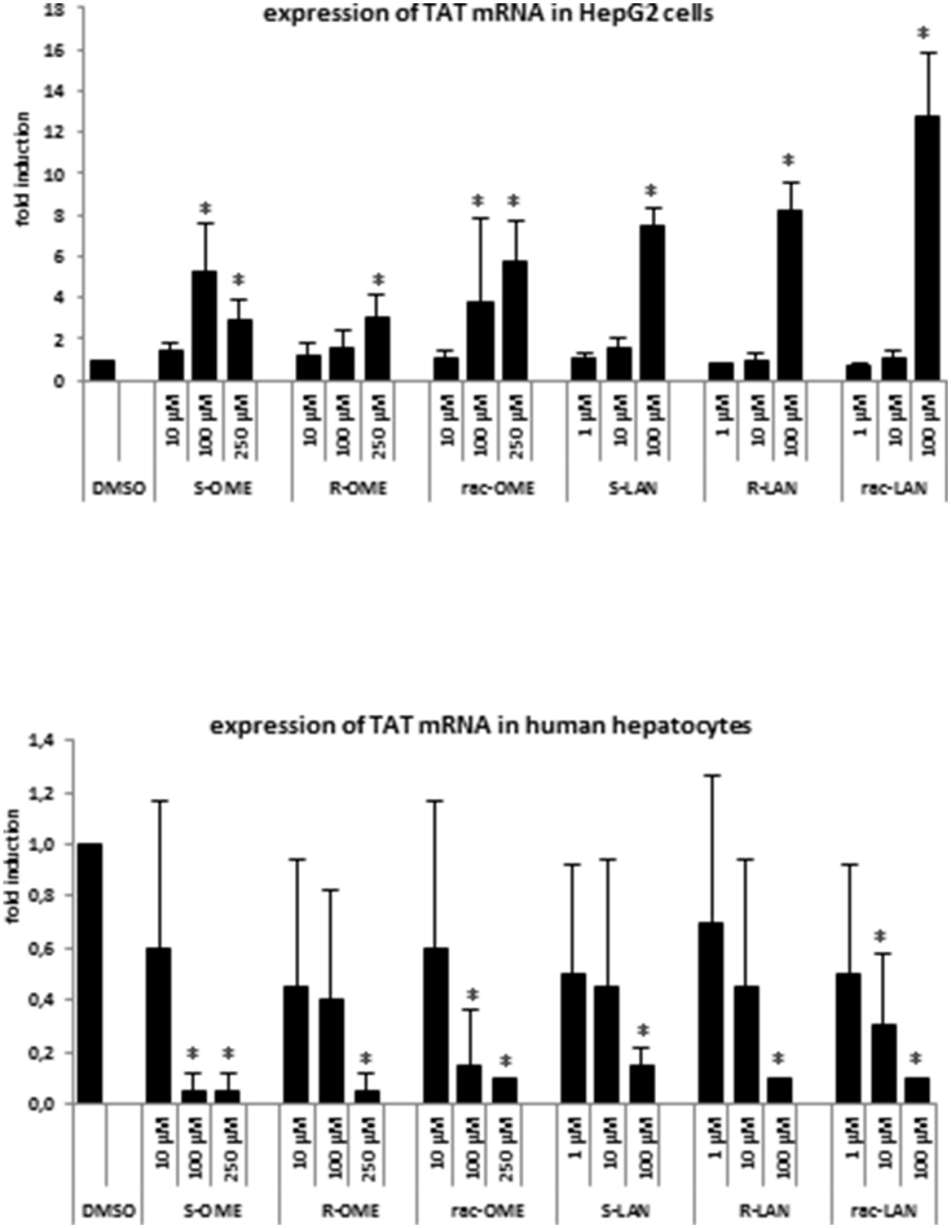
Effects of omeprazole and lansoprazole enantiomers on tyrosine aminotransferase TAT mRNA expression in HepG2 cells and human hepatocytes. Experiments were performed in two consecutive passages of HepG2 cells and in primary human hepatocytes culture Hep220770. Cells were incubated for 24 h with vehicle (DMSO; 0.1% v/v), omeprazole (S-, R-, rac-; 10 µM, 100 µM, 250 µM) and lansoprazole (S-, R-, rac-; 1 µM, 10 µM, 100 µM). Representative RT-PCR analyses of TAT mRNA are shown. The data are the mean ± SD from triplicate measurements and are expressed as a fold induction over vehicle-treated cells. The data were normalized to GAPDH mRNA levels. An asterisk (*) indicates that the value is significantly different from the activity of vehicle-treated cells.

In final series of experiments, we examined the effects of OME and LAN on the expression of tyrosinaminotransferase TAT, a prototypical and exclusive target gene for GR. All forms of OME and LAN induced TAT mRNA in HepG2 cells incubated for 24 h with tested compounds, implying an activation of GR (Figure 5; upper panel). Culture media for human hepatocytes is supplemented with dexamethasone in concentration that activates GR, therefore, we evaluated the effects of OME and LAN in antagonist mode. The expression of TAT mRNA in human hepatocytes was decreased by all forms of OME and LAN, indicating antagonistic effects of OME and LAN against GR (Figure 5; lower panel).

## Discussion

In the present paper we demonstrate that lansoprazole and omeprazole enantiomers differentially induce CYP3A4 expression in HepG2 cells and human hepatocytes, via GR-PXR-CYP3A4 axis. Taking in account the activation of AhR and induction of CYP1A1 and CYP1A2 enzymes by benzimidazole drugs, our findings bring additional information on possible drug-drug interactions and side effects of omeprazole and lansoprazole. Both compounds induced CYP3A4 in two *in vitro* models, however, their effects were qualitatively and quantitatively different for each compound, enantiomer and experimental model. In human cancer hepatic cell line HepG2, lansoprasole but not omeprazole induced CYP3A4 mRNA and protein, and the induction of CYP3A4 protein was enantiospecific with much stronger effects for S-LAN. Interestingly, we did not observe induction of CYP3A4 mRNA in intestinal human cancer cell line LS174T by neither compound tested. This difference may reflect tissue-specific regulation of CYP3A4 or cell-specific metabolism of benzimidazoles. Indeed, in our previous work, we have observed high constitutive level of CYP3A4 protein in LS174T cells, and no further induction by rifampicin, unlike in HepG2 cells [12]. Expression of CYP3A4 in primary human hepatocytes is affected not only by maternal compounds, but also by their metabolites, since human hepatocytes are highly metabolically competent *in vitro* model. For instance, it was demonstrated that omeprazole and its metabolite omeprazole sulphide display different molecular effects towards human AhR, and that conversion between drug and its metabolite is catalyzed by CYP3A4, implying a cross talk AhR-PXR in human hepatocytes [14]. Therefore, the effects of benzimidazoles in human hepatocytes were different from their effects in HepG2 cells. All forms of omeprazole and lansoprazole induced CYP3A4 mRNA and protein in two human hepatocytes cultures. The quantitative profiles of CYP3A4 induction by individual forms of omeprazole and lansoprazole exerted enantiospecific patterns. Expression of CYP3A4 in human cells is transcriptionally regulated mainly PXR, but also by other xenoreceptors (e.g. CAR), nuclear receptors (e.g. VDR) and steroid receptors (e.g. GR). In addition, the role of GR in the expression of CYP3A4 is very complex and comprises several mechanisms [15]. We used gene reporter assays in transiently and stably transfected human cell lines, to assess the effects of omeprazole and lansoprazole on transcriptional activities of PXR and GR. We found that both omeprazole and lansoprazole influence basal and ligand-activated PXR transcriptional activity, and that the effects of omeprazole but not lansoprazole were enantiospecific. Concentrations of omeprazole and lansoprazole were chosen based on pharmacokinetics data in humans. There are variations in c_MAX_ between: (i) extensive and poor metabolizers; (ii) elderly, children and adult patients; (iii) S- and R- enantiomers. Therapeutic plasma concentrations for omeprazole [16] and lansoprazole [17] are approx. 0.15–11.6 µM and 1.83–6.02 µM, respectively. The concentrations are up to five times higher that therapeutic ones, in case of overdose, which is frequent. Therefore, S-omeprazole and both enantiomers of lansoprazole activated PXR in clinically relevant concentrations. Lansoprazole but not omeprazole exerted partial agonist activity towards GR, i.e. it slightly increased basal and ligand inducible GR transcriptional activity, but these effects were not enantiospecific. In conclusion, we show that omeprazole and lansoprazole induce CYP3A4 in human cells through PXR- and GR-mediated regulation, with enantiospecific patterns. Induction of CYP3A4 by omeprazole involved probably PXR while induction by lansoprazole both PXR and GR receptors. The data presented here might be of toxicological and clinical importance.
